# Spectrophotometric Quantitation of Metformin in Bulk Drug and Pharmaceutical Formulations using Multivariate Technique

**DOI:** 10.4103/0250-474X.56022

**Published:** 2009

**Authors:** M. S. Arayne, Najma Sultana, M. H. Zuberi, F. A. Siddiqui

**Affiliations:** Department of Chemistry, University of Karachi, Karachi-75270, Pakistan; 1 Research Institute of Pharmaceutical Sciences, Faculty of Pharmacy, University of Karachi, Karachi-75270, Pakistan

**Keywords:** UV spectrophotometry, metformin, pharmaceutical analysis, biguanide derivative

## Abstract

A sensitive and accurate UV spectrophotometric method with multivariate calibration technique for the determination of metformin hydrochloride in bulk drug and different pharmaceutical formulations has been described. This technique is based on the use of the linear regression equations by using relationship between concentration and absorbance at five different wavelength. The results were treated statistically and were found highly accurate, precise and reproducible. The method is accurate, precise (% recovery 102.50±0.063, CV≤0.56, r =0.997) and linear within the range 1-10 μg/ml. There was no interference from the excipients i.e Povidone K 30, magnesium stearate, lactose and hydroxypropylmethylcellulose. This statistical approach gives optimum results for the eliminating fluctuations coming from instrumental or experimental conditions.

Metformin hydrochloride (N, N-dimethylimidodicarbonimidicdiamide hydrochloride) is a biguanide prescribed for the treatment of type II diabetes mellitus, and is the drug of choice in obese patients. It increases glucose transport across the cell membrane in skeletal muscles and it can inhibit the formation of advanced glycosylation end-products.

The reported methods for determining metformin alone[1], in multicomponent dosage forms[2,3] in combined dosage forms[4–6], in human serum[7–10] were either by HPLC, gas chromatography[11], capillary electrophoresis[12,13], NMR spectrometry[12], fluorimetry[14], potentiometry[14], PVC membrane sensor[15,16], conductometry[17,18] and NIR spectroscopy[19]. Most of these are either time consuming; involve expensive instrumentation or the use of excess organic solvents. There is no direct UV spectrophotometric method reported in the literature for the estimation of metformin. Those reported are either based on the formation of charge transfer complex or by derivative spectroscopy[18,20]. A method for the determination of levofloxacin by RP-HPLC using multivariate calibration technique has been reported[21].

The proposed method is based on the direct determination of metformin with a high degree of accuracy and sensitivity. The method is easy, least expensive and is applicable to the bulk drug and dosage formulations[21]. This paper describes the application of UV spectral multivariate calibration technique having simple mathematical content for the quantitative determination of metformin hydrochloride in pharmaceutical formulation.

The basis of this method i.e. multivariate spectral calibration contains the use of linear regression functions obtained at five different wavelengths set.[21] This approach is based on the reduction of multi-linear regression functions to univariate data set, which provides more sensitive determination than the classical UV method. In case of single wavelength UV spectrophotometry, some errors may occur because of instrumental variations and other sources.

Under optimized conditions the applied statistical method provides considerable resolving power, sensitivity, rapidity and low cost for the quantitative analysis, quality control and routine analysis of subject compounds. The mathematical algorithm of this approach is based on following summation of multivariate to univariate data sets.

If the absorbance of an analyte (Z) is measured at five wavelengths set (λ = 228, 230, 232, 234 and 236 nm), straight line equation can be written as; A_λ_ = a×(Cz+k).. (1), where A_λ_ represent the absorbance of the analyte, A is the slope and k is the intercept of linear regression function of the analyte. C_Z_ represents the concentration of analyte. At five selected wavelengths, the equation system can also be summed as; A_T_ = a×(C_Z_ + b)×(C_Z_ +c)×(C_Z_ + d)×(C_Z_ +e)×(C_Z_ +K_T_).. (2), which can be simplified to A_T_ = C_Z_ (a+b+c+d+e)+K_T_.. (3), where a, b, c, d, e are the slopes, A_T_ and K_T_ represents the sum of absorbance obtained and sum of intercepts of regression equations at five-wavelength set respectively. The concentration of the Z analyte in a mixture can be calculated by using the Eqn. C_Z_ = A_T_–K_T_/(a+b+c+d+e).. (4).

Metformin hydrochloride active was supplied by Merck Marker (Pakistan). It was tested for purity by measuring its melting point and IR spectra and no further purification were carried out. De-ionized water was used for the preparation of different dilutions. The commercial pharmaceutical formulations Glucophage® tablets (Merck Marker, Pakistan, Batch no. 1700 WHS), Neodipar® tablets (Sanophy Aventis, Pakistan, Batch No. E069) were obtained from local Pharmacies. They had an expiry of not less than 365 days at the time of study.

Spectrophotometric measurements were carried out using Shimadzu UV-1601 model UV-VIS spectrophotometer with 2 nm slit width, 1 cm quartz cell. A UVPC Personal Spectroscopy software version 3.91 was used for instrument control, data acquisition and Statistica 7 release used for data analysis.

Standard stock solution of metformin hydrochloride reference standard (100 μg/ml) in 100 ml calibrated flask was prepared in distilled water. A validation set consisting of 13 solutions in working range of 0.1-10 μg/ml were freshly prepared and scanned in the UV region. The absorption maxima observed at 232 nm was recorded and plotted against concentration, which followed the Beer and Lambert's law and gave a straight line (r=0.999). In order to improve this correlation and minimize instrumental fluctuations, absorbance of these solutions were measured over a range surrounding 232 nm i.e., 228, 230, 232, 234 and 236 nm.

Twenty metformin hydrochloride tablets were powdered in a mortar and an amount equivalent of 10 mg of drug was dissolved in 100 ml deionized water to make a solution (100 μg/ml), which was further diluted in the working range of 1-10 μg/ml. Absorbance versus concentration was plotted which gave straight line.

This study showed the applicability of multivariate linear regression approach to the UV data obtained at different wavelengths for the better calibration and tablet analysis. Statistically, the use of infinite number of data measured for a sample analysis makes the results closer to the real result. Since, metformin yields a characteristic curve when scanned in the ultraviolet range.

The five linear regression functions at the wavelengths of 228, 230, 232, 234 and 236 nm for reference standard and tablets were calculated using relationships between the absorbance and concentration. The unknown concentration of Metformin hydrochloride in tablets of two different brands was determined by the Eqn. 4 using the sum of absorbance obtained at above wavelengths for samples (Tables 1 and 2).

**TABLE 1:** CONCENTRATION FOUND IN METFORMIN IN NEODIPAR TABLETS

Concentration μg/ml	Wavelength (nm)					

	228	230	232	234	236	Multi UV^a^
1	1.01	1.04	1.06	1.08	1.08	0.9
2	2.04	2.07	2.08	2.08	2.07	2.1
3	3.08	3.14	3.17	3.18	3.18	3.0
4	4.04	4.29	4.31	4.30	4.28	4.1
5	5.43	5.47	5.49	5.50	5.48	5.2
6	6.14	6.07	6.10	6.12	6.01	5.8
7	7.10	7.21	7.27	7.27	7.22	7.0
8	8.30	8.34	8.34	8.28	8.22	8.0
9	9.53	9.52	9.49	9.46	9.44	9.1
10	10.30	10.75	10.30	10.27	10.21	10.0

a multivariate UV data, concentration in μg/ml

**TABLE 2:** CONCENTRATION FOUND IN METFORMIN IN GLUCOPHAGE TABLETS

Concentration μg/ml	Wavelength (nm)					

	228	230	232	234	236	Multi UV^a^
1	1.00	1.03	1.06	1.06	1.06	0.9
2	1.97	2.03	2.08	2.09	2.10	2.1
3	3.02	3.10	3.15	3.18	3.19	3.0
4	4.20	4.29	4.34	4.36	4.37	4.1
5	5.15	5.26	5.32	5.36	5.37	5.0
6	6.15	6.24	6.17	6.42	6.35	5.9
7	7.10	7.21	7.28	7.32	7.33	6.9
8	8.34	8.42	8.45	8.45	8.22	8.0
9	9.50	9.58	9.62	9.63	9.58	9.1
10	10.39	10.52	10.59	10.60	10.54	10.0

a multivariate UV data, concentration in μg/ml

The method was linear in the range of 1-10 μg/ml. Under the experimental conditions describe above, linear regression equation (intercept and slope) for metformin hydrochloride was established. The high values of the correlation coefficient and the values of Y-intercept close to zero indicate good linearity of the calibrations (Table 3).

**TABLE 3:** REGRESSION CHARACTERISTICS OF PROPOSED METHOD

Drug	Wavelength (nm)	Regression Equation	r	SE*	SEE**	LOD	LOQ
Metformin	228	A = 0.0779 Cx+0.0088	0.999	0.05	0.114	0.253	0.845
	230	A= 0.0814 Cx+0.0069	0.998	0.039	0.085	0.223	0.744
	232	A = 0.0832 Cx+0.0060	0.999	0.035	0.077	0.253	0.745
	234	A = 0.0821 Cx+0.0044	0.998	0.033	0.071	0.223	0.73
	236	A = 0.0789 Cx+0.0038	0.997	0.029	0.063	0.215	0.718
Glucophage	228	A = 0.0796 Cx+0.0177	0.999	0.05	0.072	0.195	0.649
(Tablet)	230	A = 0.0849 Cx+0.0134	0.999	0.051	0.073	0.184	0.614
	232	A = 0.0846 Cx+0.0210	0.997	0.052	0.074	0.179	0.596
	234	A = 0.0834 Cx+0.0193	0.999	0.051	0.073	0.178	0.593
	236	A= 0.0798 Cx+0.0176	0.999	0.052	0.075	0.187	0.622
Neodipar	228	A= 0.0796 Cx+0.0177	0.999	0.098	0.14	0.264	0.88
(Tablet)	230	A= 0.0849 Cx+0.0134	0.998	0.109	0.157	0.204	0.681
	232	A = 0.0846 Cx+0.0210	0.998	0.085	0.12	0.189	0.55
	234	A= 0.0834 Cx+0.0193	0.998	0.107	0.152	0.165	0.478
	236	A= 0.0798 Cx+0.0176	0.999	0.112	0.159	0.143	0.431

* SE is standard error and

** SEE is the standard error of estimation

The method was validated according to International Conference on Harmonization (ICH) Q2B complete ref guidelines for validation of analytical procedures in order to determine the linearity, sensitivity, precision and accuracy. Precision of the method was determined by adding known amounts of pure drug (90, 100 and 110%) in triplicate. Table 4 summarizes the statistical results evaluated from the above observations. For the accuracy of the developed method, standard addition method was done. Different concentrations of pure drug (3.6, 4 and 4.4 μg/ml) were added to a known pre-analysed formulation sample and the total concentration was determined (Table 5). The percent recovery of the added pure drug was calculated as follows; % recovery= [(Cv−Cu)/Ca] 100, where Cv was the total drug concentration measured after standard addition, Cu, drug concentration in the formulation and Ca, drug concentration added to formulation.

**TABLE 4:** EVALUATION OF PRECISION OF THE PROPOSED METHOD IN PURE DRUG SUBSTANCE

Amount of drug added (mg/100ml)	Individual amounts found (mg/100 ml)	(SD)^a^	(CV)	Confidence limits^b^
27.1	27.24	0.152	0.56	26.51-27.52
27.12	26.95				
26.89	26.85				
mean	27.01			
30.14	30.07	0.158	0.53	29.52-30.29
30.06	29.9			
29.8	29.76			
mean	29.91			
33.34	33.21	0.139	0.42	32.75-33.40
33.2	33.08			
33.26	32.95			
mean	33.08			

a n=6; SD is the standard deviation; CV is coefficient of variation

b Confidence limits at P=0.95 and two degrees of freedom.

**TABLE 5:** ACCURACY OF THE PROPOSED METHOD (STANDARD ADDITION TECHNIQUE)

Conc. of drug in formulations (μg/ml)	Conc. of pure drug added (μg/ml)	Total conc. of drug found (μg/ml)	% Analytical recov. (±SD)	*CL
5	3.6	8.69	102.50±0.063	03.59-03.77
5	4	9.12	103.00±0.084	04.02-04.18
5	4.4	9.52	102.72±0.084	04.18-04.81

Each value is the result of three separate determinations.

* Confidence limits at P=0.95 and two degrees of freedom.

LOD is the lowest concentration of an analyte that an analytical process can reliably detect (0.082 μg/ml). LOQ is defined as the lowest concentration of the standard that can be measured (0.25 μg/ml). The LOD and LOQ were calculated according to ICH guideline as LOD = 3.3σ/S and LOQ = 10σ/S, where σ is the standard deviation of the lowest standard concentration and S is the slope of the standard curve.

Concept behind this effort was to minimize the uncertain hindrances caused during the observation. In this Paper, statistical analysis with multivariate spectral technique was used. The data obtained for the estimation of metformin in bulk and drug formulation evidenced the high level accuracy and precision after multivariate calibration. Percent recovery and found concentration of active ingredient in pharmaceutical formulations showed that the amount of drug present is consistent with the label claim. Hence, this method is very useful with very simple mathematical contents, is more reliable than the other spectrophotometric methods and strongly recommends the application in calibration models for a routine analysis.
